# The genome sequence of the European flat oyster, *Ostrea edulis *(Linnaeus, 1758)

**DOI:** 10.12688/wellcomeopenres.19916.1

**Published:** 2023-12-05

**Authors:** Patrick Adkins, Rob Mrowicki, Marine Biological Association Genome Acquisition Lab, Darwin Tree of Life Barcoding collective, Wellcome Sanger Institute Tree of Life programme, Wellcome Sanger Institute Scientific Operations: DNA Pipelines collective, Tree of Life Core Informatics collective, Darwin Tree of Life Consortium

**Affiliations:** 1 The Marine Biological Association, Plymouth, England, UK

**Keywords:** Ostrea edulis, European flat oyster, genome sequence, chromosomal, Ostreida

## Abstract

We present a genome assembly from an individual *Ostrea edulis* (the European flat oyster; Mollusca; Bivalvia; Ostreida; Ostreidae). The genome sequence is 894.8 megabases in span. Most of the assembly is scaffolded into 10 chromosomal pseudomolecules. The mitochondrial genome has also been assembled and is 16.35 kilobases in length.

## Species taxonomy

Eukaryota; Metazoa; Eumetazoa; Bilateria; Protostomia; Spiralia; Lophotrochozoa; Mollusca; Bivalvia; Autobranchia; Pteriomorphia; Ostreida; Ostreoidea; Ostreidae; *Ostrea*; *Ostrea edulis* (Linnaeus, 1758) (NCBI:txid37623).

## Background

*Ostrea edulis*, the European flat oyster, is a variably round to oval or pear-shaped bivalve that grows to approximately 110 mm in diameter. It has a cream or off-white shell, which is usually discoloured grey or covered in epiphytic growth. It can be found living in the low intertidal and shallow shelf seas on bottoms of firm mud and hard silt, particularly associated with estuarine habitats ( Perry & Jackson, 2017). Ecologically, it serves as a keystone species and habitat engineer, forming extensive beds, which are important habitats and feeding grounds for many species, thus greatly increasing biodiversity where it is present ( Bennema *et al.*, 2020; Coen *et al.*, 2007; Grabowski & Peterson, 2007).

*Ostrea edulis* was an abundant and important species around the UK, likely since prehistory ( Gutiérrez-Zugasti *et al.*, 2011; Helmer *et al.*, 2019). It has supported a large fishery since the 13th century, which peaked in the 19th century. In the year 1864 alone, 700 million oysters were caught in London ( Lotze, 2007; Philpots, 1891; Rodriguez-Perez *et al.*, 2019). However, the population started to decline since the late 19th and early 20th century due to a combination of pressures ( Pogoda, 2019; South of England Oyster Company, 1865; Yonge, 1960). Factors such as overexploitation ( Colsoul *et al.*, 2021), the introduction of invasive species such as *Magallana gigas* ( Guy *et al.*, 2018) and *Crepidula fornicata* ( Preston *et al.*, 2020), disease ( Culloty & Mulcahy, 2007), habitat destruction ( Berghahn & Ruth, 2005), and pollution ( Thouzeau *et al.*, 2003), have all led to a rapid decline across the UK. Estimates of the remaining native oyster population range from 15% to 1% ( Allison, 2019; Beck *et al.*, 2011; Helmer *et al.*, 2019; Zu Ermgassen *et al.*, 2012). As a result, heterospecific *O. edulis* beds are now rare in the UK, and have vanished from much of its original range or been replaced by *Magallana gigas* ( Haelters & Kerckhof, 2009).

*Ostrea edulis* has been the target of reintroduction and restocking projects and conservation efforts ( Bromley *et al.*, 2016; Rodriguez-Perez *et al.*, 2019). It is currently listed as threatened or declining by OSPAR ( Haelters & Kerckhof, 2009).

The genome of *Ostrea edulis* has been previously sequenced ( Gundappa *et al.*, 2022). In this work, as a part of the Darwin Tree of Life programme, we present a chromosomally-complete sequence of the species, based on a specimen collected from Devon, UK.

## Genome sequence report

**Figure 1.**
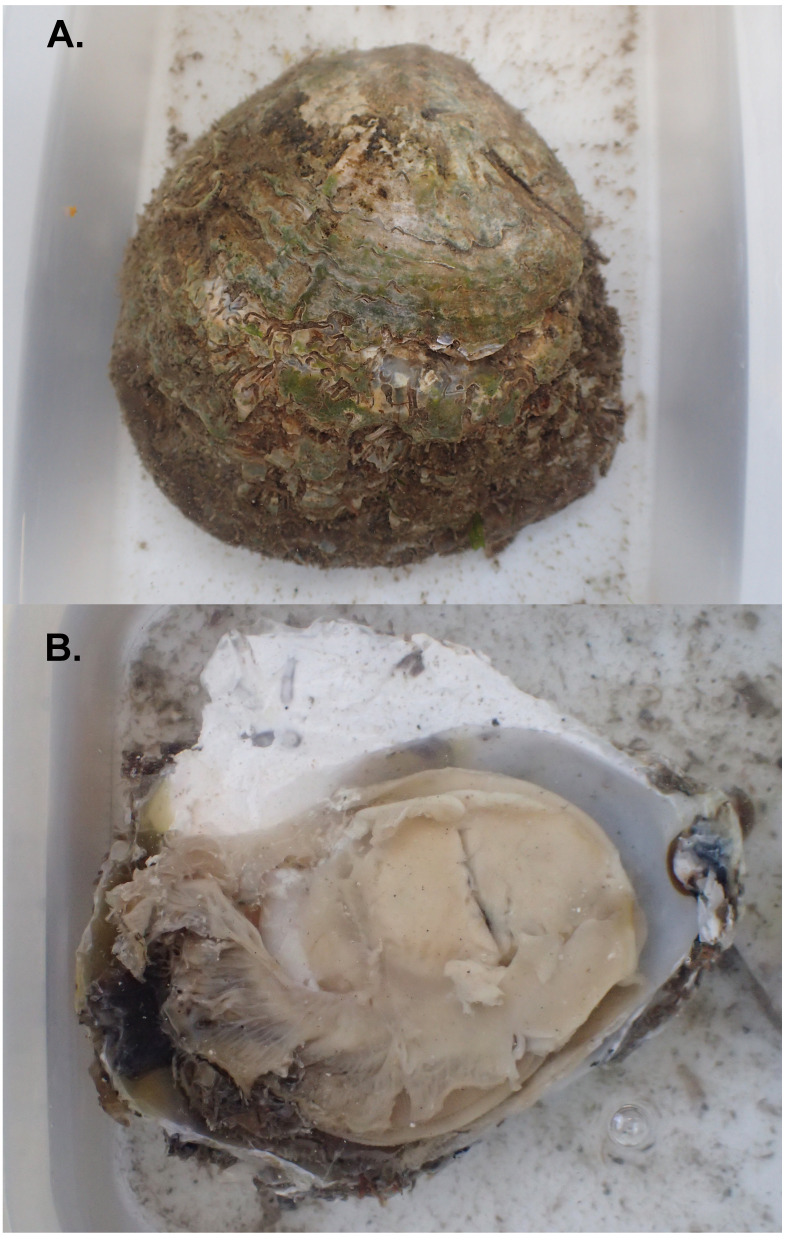
Photographs of the *Ostrea edulis* (xbOstEdul1) specimen used for genome sequencing.

The genome was sequenced from one *Ostrea edulis* ( Figure 1) collected from Western Point, Oreston, Devon, UK (50.36, –4.11). A total of 38-fold coverage in Pacific Biosciences single-molecule HiFi long reads was generated. Primary assembly contigs were scaffolded with chromosome conformation Hi-C data. Manual assembly curation corrected 94 missing joins or mis-joins and removed 33 haplotypic duplications, reducing the assembly length by 70.95% and the scaffold number by 48.54%, and increasing the scaffold N50 by 0.35%.

**Figure 2.**
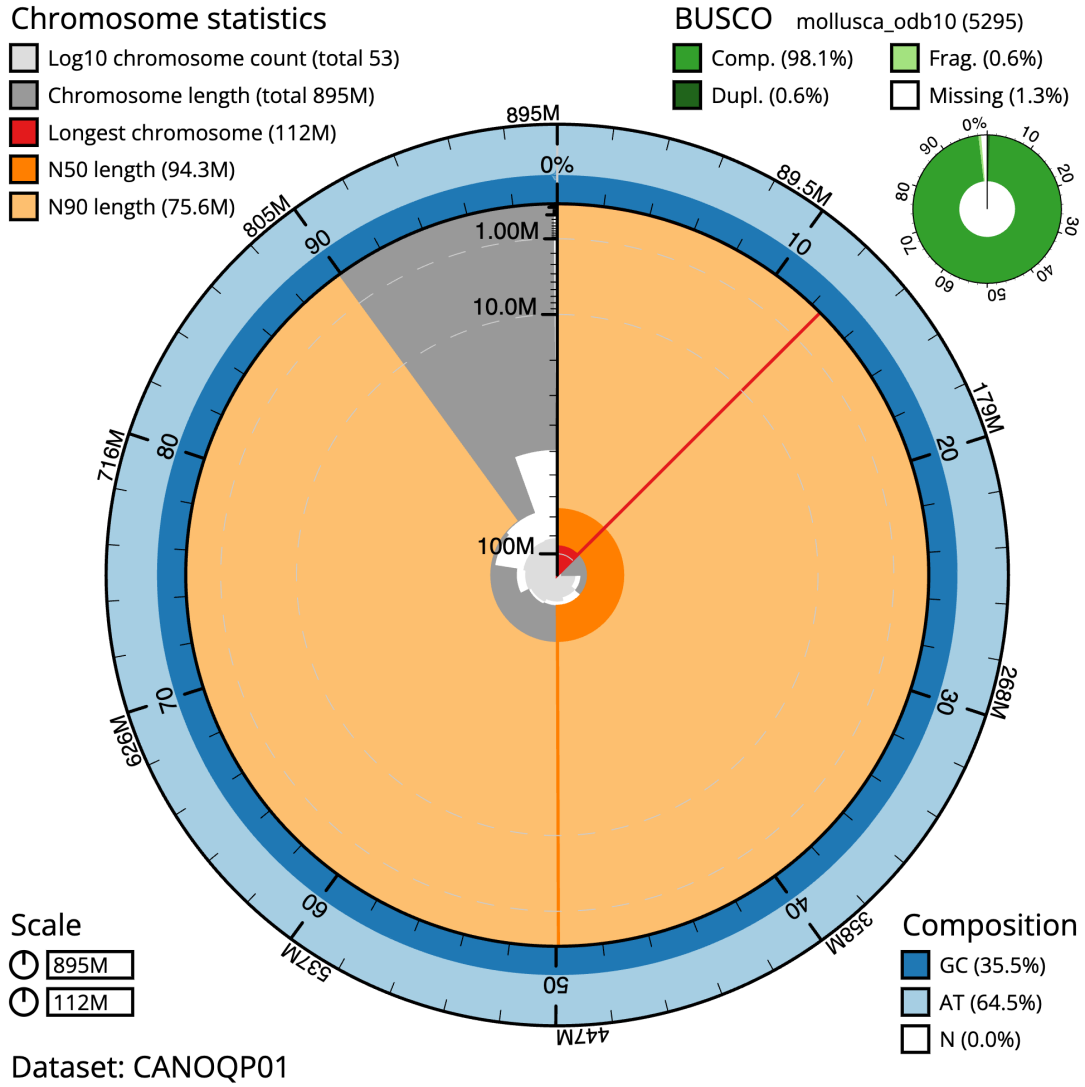
Genome assembly of *Ostrea edulis*, xbOstEdul1.1: metrics. The BlobToolKit Snailplot shows N50 metrics and BUSCO gene completeness. The main plot is divided into 1,000 size-ordered bins around the circumference with each bin representing 0.1% of the 894,803,243 bp assembly. The distribution of scaffold lengths is shown in dark grey with the plot radius scaled to the longest scaffold present in the assembly (112,480,954 bp, shown in red). Orange and pale-orange arcs show the N50 and N90 scaffold lengths (94,306,699 and 75,578,129 bp), respectively. The pale grey spiral shows the cumulative scaffold count on a log scale with white scale lines showing successive orders of magnitude. The blue and pale-blue area around the outside of the plot shows the distribution of GC, AT and N percentages in the same bins as the inner plot. A summary of complete, fragmented, duplicated and missing BUSCO genes in the mollusca_odb10 set is shown in the top right. An interactive version of this figure is available at https://blobtoolkit.genomehubs.org/view/xbOstEdul1.1/dataset/CANOQP01/snail.

The final assembly has a total length of 894.8 Mb in 52 sequence scaffolds with a scaffold N50 of 94.3 Mb ( Table 1). Most (99.79%) of the assembly sequence was assigned to 10 chromosomal-level scaffolds. Chromosome-scale scaffolds confirmed by the Hi-C data are named in order of size ( Figure 2– Figure 5; Table 2). While not fully phased, the assembly deposited is of one haplotype. Contigs corresponding to the second haplotype have also been deposited. The mitochondrial genome was also assembled and can be found as a contig within the multifasta file of the genome submission.

**Table 1.** Genome data for *Ostrea edulis*, xbOstEdul1.1.

Project accession data		
Assembly identifier	xbOstEdul1.1	
Species	*Ostrea edulis*	
Specimen	xbOstEdul1	
NCBI taxonomy ID	37623	
BioProject	PRJEB57260	
BioSample ID	SAMEA12219414	
Isolate information	xbOstEdul1: muscle (DNA sequencing and Hi-C data)	
Assembly metrics *		*Benchmark*
Consensus quality (QV)	59.4	*≥ 50*
*k*-mer completeness	100%	*≥ 95%*
BUSCO **	C:98.1%[S:97.5%,D:0.6%], F:0.6%,M:1.3%,n:5,295	*C ≥ 95%*
Percentage of assembly mapped to chromosomes	99.79%	*≥ 95%*
Sex chromosomes	-	*localised homologous pairs*
Organelles	Mitochondrial genome assembled	*complete single alleles*
Raw data accessions		
PacificBiosciences SEQUEL II	ERR10466794, ERR10466793	
Hi-C Illumina	ERR10466803	
PolyA RNA-Seq Illumina	ERR10890708	
Genome assembly		
Assembly accession	GCA_947568905.1	
*Accession of alternate haplotype*	GCA_947568865.1	
Span (Mb)	894.8	
Number of contigs	658	
Contig N50 length (Mb)	2.4	
Number of scaffolds	52	
Scaffold N50 length (Mb)	94.3	
Longest scaffold (Mb)	112.5	

* Assembly metric benchmarks are adapted from column VGP-2020 of “Table 1: Proposed standards and metrics for defining genome assembly quality” from ( Rhie *et al.*, 2021). ** BUSCO scores based on the mollusca_odb10 BUSCO set using v5.3.2. C = complete [S = single copy, D = duplicated], F = fragmented, M = missing, n = number of orthologues in comparison. A full set of BUSCO scores is available at https://blobtoolkit.genomehubs.org/view/xbOstEdul1.1/dataset/CANOQP01/busco

**Table 2.** Chromosomal pseudomolecules in the genome assembly of *Ostrea edulis*, xbOstEdul1.

INSDC accession	Chromosome	Length (Mb)	GC%
OX387704.1	1	112.48	35.5
OX387705.1	2	109.48	35.5
OX387706.1	3	98.27	35.5
OX387707.1	4	94.44	35.5
OX387708.1	5	94.31	35.5
OX387709.1	6	93.53	35.5
OX387710.1	7	88.74	35.5
OX387711.1	8	77.13	35.5
OX387712.1	9	75.58	35.5
OX387713.1	10	48.99	35.5
OX387714.1	MT	0.02	35.0

**Figure 3.**
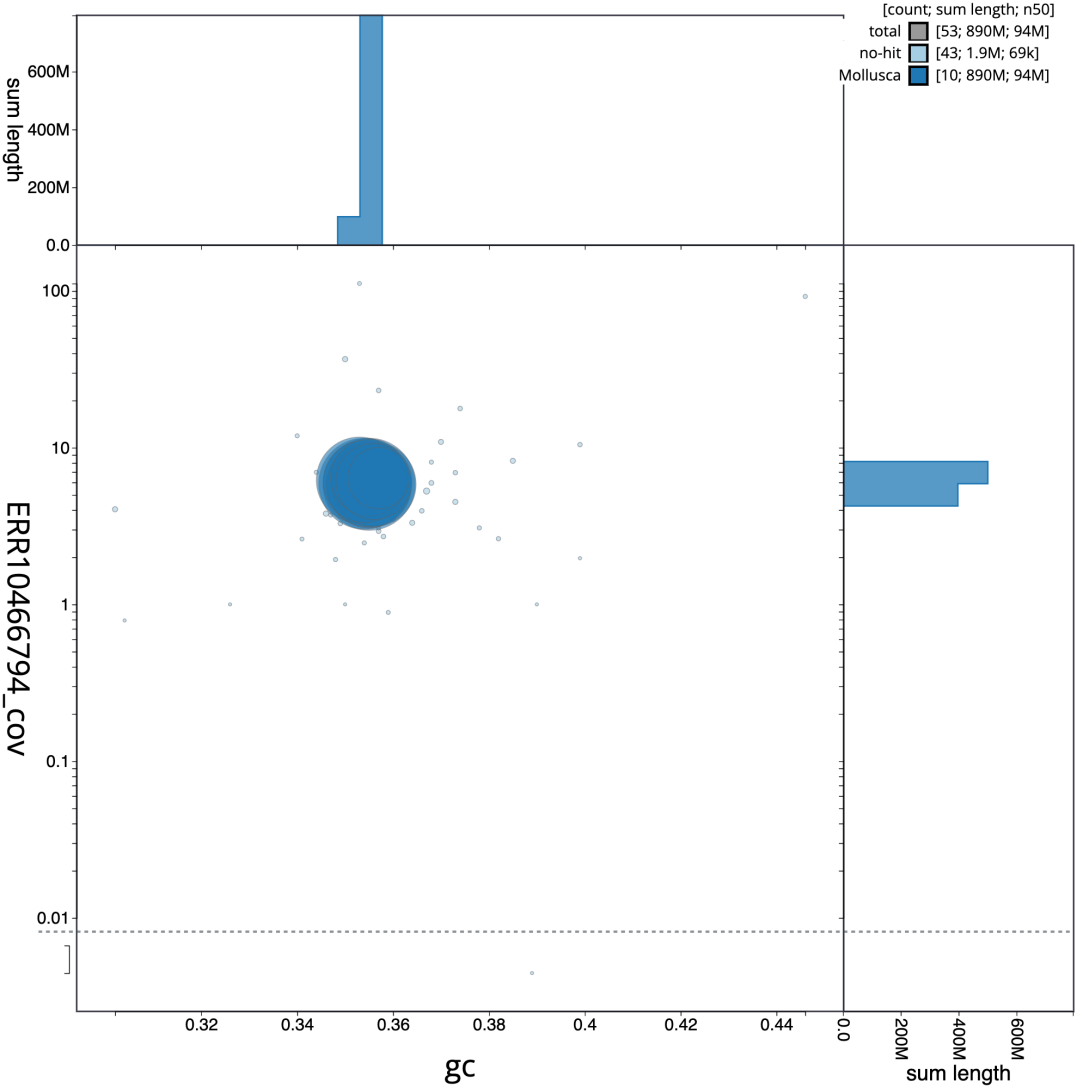
Genome assembly of *Ostrea edulis*, xbOstEdul1.1: BlobToolKit GC-coverage plot. Scaffolds are coloured by phylum. Circles are sized in proportion to scaffold length. Histograms show the distribution of scaffold length sum along each axis. An interactive version of this figure is available at https://blobtoolkit.genomehubs.org/view/xbOstEdul1.1/dataset/CANOQP01/blob.

**Figure 4.**
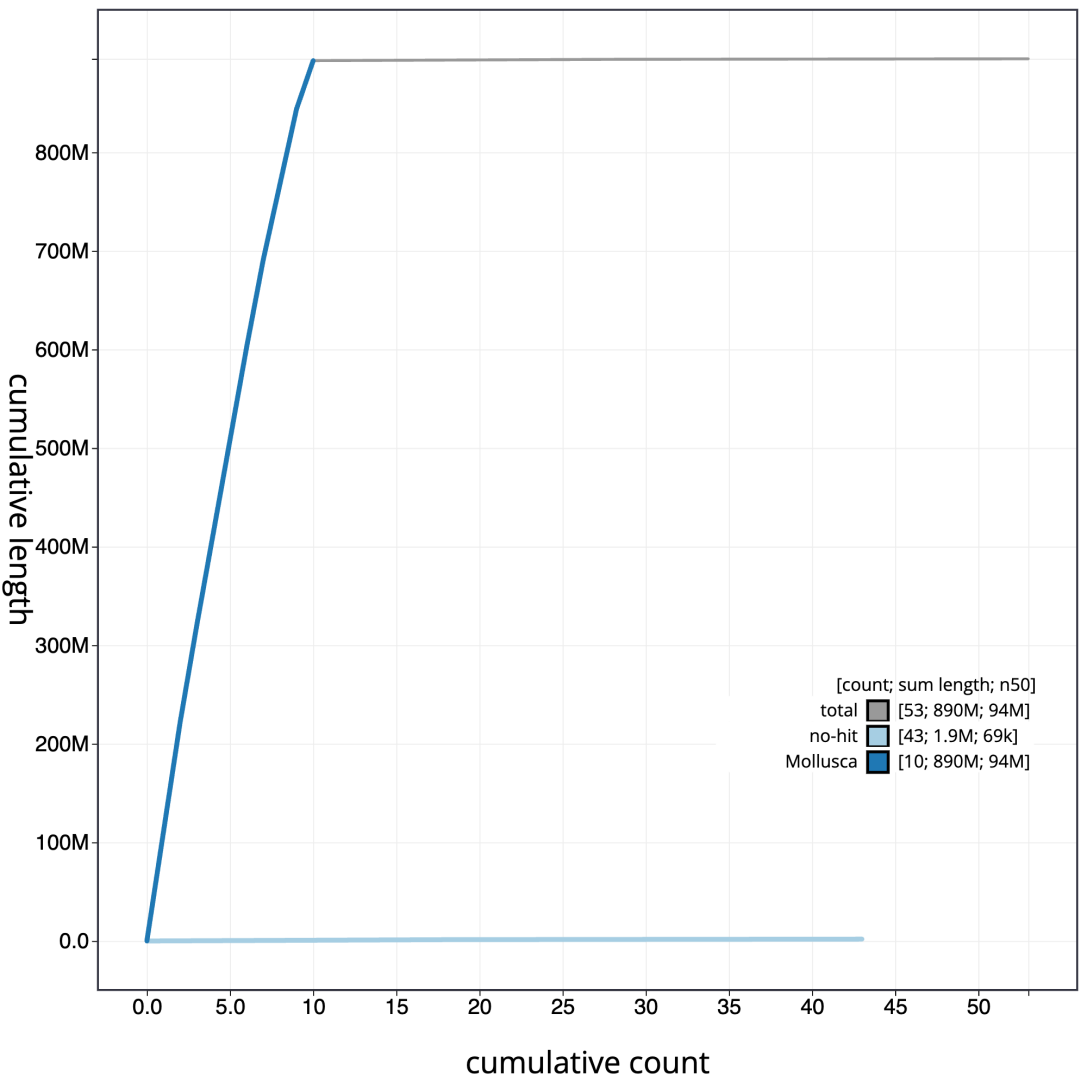
Genome assembly of *Ostrea edulis*, xbOstEdul1.1: BlobToolKit cumulative sequence plot. The grey line shows cumulative length for all scaffolds. Coloured lines show cumulative lengths of scaffolds assigned to each phylum using the buscogenes taxrule. An interactive version of this figure is available at https://blobtoolkit.genomehubs.org/view/xbOstEdul1.1/dataset/CANOQP01/cumulative.

**Figure 5.**
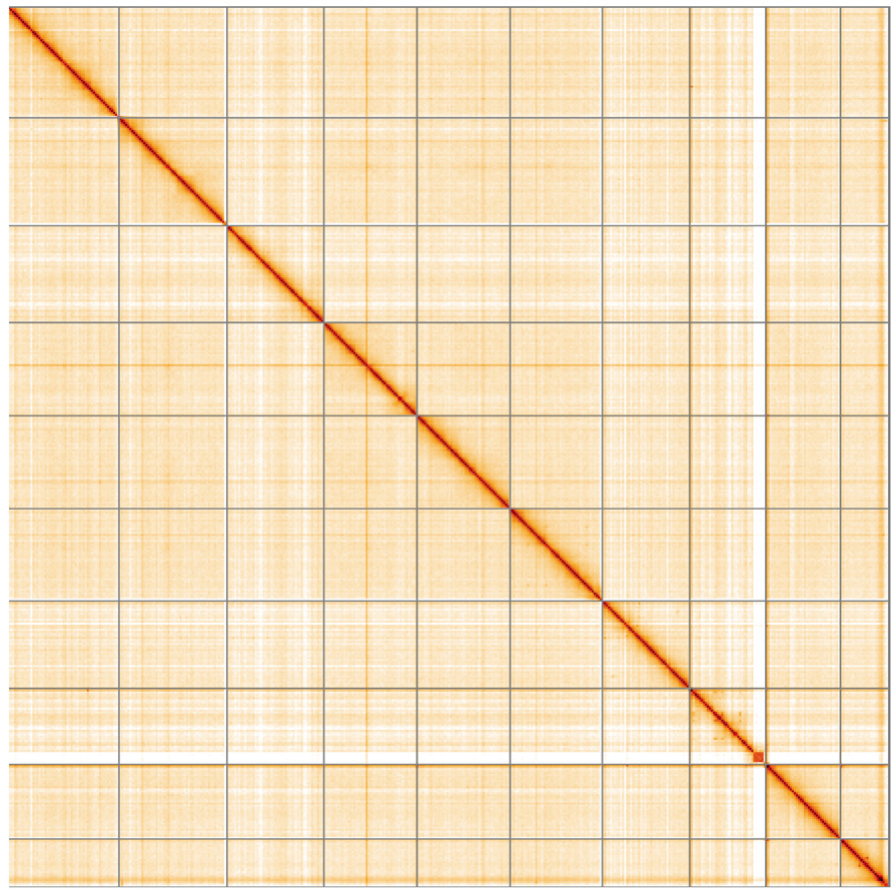
Genome assembly of *Ostrea edulis*, xbOstEdul1.1: Hi-C contact map of the xbOstEdul1.1 assembly, visualised using HiGlass. Chromosomes are shown in order of size from left to right and top to bottom. An interactive version of this figure may be viewed at https://genome-note-higlass.tol.sanger.ac.uk/l/?d=ZheqF50kSq23AUjYR99Udw.

The estimated Quality Value (QV) of the final assembly is 59.4 with *k*-mer completeness of 100%, and the assembly has a BUSCO v5.3.2 completeness of 98.1% (single = 97.5%, duplicated = 0.6%), using the mollusca_odb10 reference set ( *n* = 5,295).

Metadata for specimens, spectral estimates, sequencing runs, contaminants and pre-curation assembly statistics can be found at https://links.tol.sanger.ac.uk/species/37623.

## Methods

### Sample acquisition and nucleic acid extraction

A *Ostrea edulis* (specimen ID MBA-210429-001A, individual xbOstEdul1) was collected from Western Point, Oreston, Devon, UK (latitude 50.36, longitude –4.11) on 2021-04-29. The specimen was collected by hand and placed in a container. The specimen was collected and identified by Patrick Adkins and Rob Mrowicki (both Marine Biological Association), and preserved in liquid nitrogen. 

DNA was extracted at the Tree of Life laboratory, Wellcome Sanger Institute (WSI). The xbOstEdul1 sample was weighed and dissected on dry ice with tissue set aside for Hi-C sequencing. Muscle tissue was cryogenically disrupted to a fine powder using a Covaris cryoPREP Automated Dry Pulveriser, receiving multiple impacts. High molecular weight (HMW) DNA was extracted using the Qiagen MagAttract HMW DNA extraction kit. HMW DNA was sheared into an average fragment size of 12–20 kb in a Megaruptor 3 system with speed setting 30. Sheared DNA was purified by solid-phase reversible immobilisation using AMPure PB beads with a 1.8X ratio of beads to sample to remove the shorter fragments and concentrate the DNA sample. The concentration of the sheared and purified DNA was assessed using a Nanodrop spectrophotometer and Qubit Fluorometer and Qubit dsDNA High Sensitivity Assay kit. Fragment size distribution was evaluated by running the sample on the FemtoPulse system.

RNA was extracted from muscle tissue of xbOstEdul1 in the Tree of Life Laboratory at the WSI using TRIzol, according to the manufacturer’s instructions. RNA was then eluted in 50 μl RNAse-free water and its concentration assessed using a Nanodrop spectrophotometer and Qubit Fluorometer using the Qubit RNA Broad-Range (BR) Assay kit. Analysis of the integrity of the RNA was done using Agilent RNA 6000 Pico Kit and Eukaryotic Total RNA assay.

### Sequencing

Pacific Biosciences HiFi circular consensus DNA sequencing libraries were constructed according to the manufacturers’ instructions. Poly(A) RNA-Seq libraries were constructed using the NEB Ultra II RNA Library Prep kit. DNA and RNA sequencing was performed by the Scientific Operations core at the WSI on Pacific Biosciences SEQUEL II (HiFi) and Illumina NovaSeq 6000 instruments. Hi-C data were also generated from muscle tissue of xbOstEdul1 using the Arima2 kit and sequenced on the Illumina NovaSeq 6000 instrument.

### Genome assembly, curation and evaluation

Assembly was carried out with Hifiasm ( Cheng *et al.*, 2021) and haplotypic duplication was identified and removed with purge_dups ( Guan *et al.*, 2020). The assembly was then scaffolded with Hi-C data ( Rao *et al.*, 2014) using YaHS ( Zhou *et al.*, 2023). The assembly was checked for contamination and corrected using the gEVAL system ( Chow *et al.*, 2016) as described previously ( Howe *et al.*, 2021). Manual curation was performed using gEVAL, HiGlass ( Kerpedjiev *et al.*, 2018) and Pretext ( Harry, 2022). The mitochondrial genome was assembled using MitoHiFi ( Uliano-Silva *et al.*, 2023), which runs MitoFinder ( Allio *et al.*, 2020) or MITOS ( Bernt *et al.*, 2013) and uses these annotations to select the final mitochondrial contig and to ensure the general quality of the sequence.

A Hi-C map for the final assembly was produced using bwa-mem2 ( Vasimuddin *et al.*, 2019) in the Cooler file format ( Abdennur & Mirny, 2020). To assess the assembly metrics, the *k*-mer completeness and QV consensus quality values were calculated in Merqury ( Rhie *et al.*, 2020). This work was done using Nextflow ( Di Tommaso *et al.*, 2017) DSL2 pipelines “sanger-tol/readmapping” ( Surana *et al.*, 2023a) and “sanger-tol/genomenote” ( Surana *et al.*, 2023b). The genome was analysed within the BlobToolKit environment ( Challis *et al.*, 2020) and BUSCO scores ( Manni *et al.*, 2021; Simão *et al.*, 2015) were calculated.

Table 3 contains a list of relevant software tool versions and sources.

**Table 3.** Software tools: versions and sources.

Software tool	Version	Source
BlobToolKit	4.1.7	https://github.com/blobtoolkit/blobtoolkit
BUSCO	5.3.2	https://gitlab.com/ezlab/busco
Hifiasm	0.16.1-r375	https://github.com/chhylp123/hifiasm
HiGlass	1.11.6	https://github.com/higlass/higlass
Merqury	MerquryFK	https://github.com/thegenemyers/MERQURY.FK
MitoHiFi	2	https://github.com/marcelauliano/MitoHiFi
PretextView	0.2	https://github.com/wtsi-hpag/PretextView
purge_dups	1.2.3	https://github.com/dfguan/purge_dups
sanger-tol/genomenote	v1.0	https://github.com/sanger-tol/genomenote
sanger-tol/readmapping	1.1.0	https://github.com/sanger-tol/readmapping/tree/1.1.0
YaHS	1.1a.2	https://github.com/c-zhou/yahs

### Wellcome Sanger Institute – Legal and Governance

The materials that have contributed to this genome note have been supplied by a Darwin Tree of Life Partner. The submission of materials by a Darwin Tree of Life Partner is subject to the **‘Darwin Tree of Life Project Sampling Code of Practice’,** which can be found in full on the Darwin Tree of Life website here. By agreeing with and signing up to the Sampling Code of Practice, the Darwin Tree of Life Partner agrees they will meet the legal and ethical requirements and standards set out within this document in respect of all samples acquired for, and supplied to, the Darwin Tree of Life Project.

Further, the Wellcome Sanger Institute employs a process whereby due diligence is carried out proportionate to the nature of the materials themselves, and the circumstances under which they have been/are to be collected and provided for use. The purpose of this is to address and mitigate any potential legal and/or ethical implications of receipt and use of the materials as part of the research project, and to ensure that in doing so we align with best practice wherever possible. The overarching areas of consideration are:

•     Ethical review of provenance and sourcing of the material

•     Legality of collection, transfer and use (national and international) 

Each transfer of samples is further undertaken according to a Research Collaboration Agreement or Material Transfer Agreement entered into by the Darwin Tree of Life Partner, Genome Research Limited (operating as the Wellcome Sanger Institute), and in some circumstances other Darwin Tree of Life collaborators.

## Data Availability

European Nucleotide Archive: *Ostrea edulis* (native oyster). Accession number PRJEB57260; https://identifiers.org/ena.embl/PRJEB57260. ( Wellcome Sanger Institute, 2022) The genome sequence is released openly for reuse. The *Ostrea edulis* genome sequencing initiative is part of the Darwin Tree of Life (DToL) project. All raw sequence data and the assembly have been deposited in INSDC databases. The genome will be annotated using available RNA-Seq data and presented through the Ensembl pipeline at the European Bioinformatics Institute. Raw data and assembly accession identifiers are reported in Table 1.

## Author information

Members of the Marine Biological Association Genome Acquisition Lab are listed here: https://doi.org/10.5281/zenodo.4783604.

Members of the Darwin Tree of Life Barcoding collective are listed here: https://doi.org/10.5281/zenodo.4893703.

Members of the Wellcome Sanger Institute Tree of Life programme are listed here: https://doi.org/10.5281/zenodo.4783585.

Members of Wellcome Sanger Institute Scientific Operations: DNA Pipelines collective are listed here: https://doi.org/10.5281/zenodo.4790455.

Members of the Tree of Life Core Informatics collective are listed here: https://doi.org/10.5281/zenodo.5013541.

Members of the Darwin Tree of Life Consortium are listed here: https://doi.org/10.5281/zenodo.4783558.

## References

[ref-1] Abdennur N Mirny LA : Cooler: Scalable storage for Hi-C data and other genomically labeled arrays. *Bioinformatics.* 2020;36(1):311–316. 10.1093/bioinformatics/btz540 31290943 PMC8205516

[ref-2] Allio R Schomaker-Bastos A Romiguier J : MitoFinder: Efficient automated large-scale extraction of mitogenomic data in target enrichment phylogenomics. *Mol Ecol Resour.* 2020;20(4):892–905. 10.1111/1755-0998.13160 32243090 PMC7497042

[ref-3] Allison S : The endangered European native oyster *Ostrea edulis* (L) and creation of Marine Conservation Zones: a win-win scenario for fisheries and conservation?University of Essex,2019. Reference Source

[ref-4] Beck MW Brumbaugh RD Airoldi L : Oyster Reefs at Risk and Recommendations for Conservation, Restoration, and Management. * BioScience.* 2011;61(2):107–116. 10.1525/bio.2011.61.2.5

[ref-5] Bennema FP Engelhard GH Lindeboom H : *Ostrea edulis* beds in the central North Sea: delineation, ecology, and restoration. *ICES J Mar Sci.* 2020;77(7–8):2694–2705. 10.1093/icesjms/fsaa134

[ref-6] Berghahn R Ruth M : The disappearance of oysters from the Wadden Sea: a cautionary tale for no-take zones. *Aquat Conserv.* 2005;15(1):91–104. 10.1002/aqc.635

[ref-7] Bernt M Donath A Jühling F : MITOS: Improved *de novo* metazoan mitochondrial genome annotation. *Mol Phylogenet Evol.* 2013;69(2):313–319. 10.1016/j.ympev.2012.08.023 22982435

[ref-8] Bromley C McGonigle C Ashton EC : Bad moves: Pros and cons of moving oysters - A case study of global translocations of *Ostrea edulis* Linnaeus, 1758 (Mollusca: Bivalvia). *Ocean & Coastal Management.* 2016;122:103–115. 10.1016/j.ocecoaman.2015.12.012

[ref-9] Challis R Richards E Rajan J : BlobToolKit - interactive quality assessment of genome assemblies. *G3 (Bethesda).* 2020;10(4):1361–1374. 10.1534/g3.119.400908 32071071 PMC7144090

[ref-10] Cheng H Concepcion GT Feng X : Haplotype-resolved *de novo* assembly using phased assembly graphs with hifiasm. *Nat Methods.* 2021;18(2):170–175. 10.1038/s41592-020-01056-5 33526886 PMC7961889

[ref-11] Chow W Brugger K Caccamo M : gEVAL — a web-based browser for evaluating genome assemblies. *Bioinformatics.* 2016;32(16):2508–2510. 10.1093/bioinformatics/btw159 27153597 PMC4978925

[ref-12] Coen LD Brumbaugh RD Bushek D : Ecosystem services related to oyster restoration. *Marine Ecology Progress Series.* 2007;341:303–307. Reference Source

[ref-13] Colsoul B Boudry P Pérez-Parallé ML : Sustainable large-scale production of European flat oyster ( *Ostrea edulis* ) seed for ecological restoration and aquaculture: a review. *Reviews in Aquaculture.* 2021;13(3):1423–1468. 10.1111/raq.12529

[ref-14] Culloty SC Mulcahy MF : *Bonamia ostreae* in the Native Oyster *Ostrea edulis*.Marine Institute.2007. Reference Source

[ref-15] Di Tommaso P Chatzou M Floden EW : Nextflow enables reproducible computational workflows. *Nat Biotechnol.* 2017;35(4):316–319. 10.1038/nbt.3820 28398311

[ref-16] Grabowski JH Peterson CH : Restoring oyster reefs to recover ecosystem services.In: Kim Cuddington, James E. Byers, William G. Wilson, and Alan Hastings (eds.) *Ecosystem engineers: plants to protists*. Elsevier,2007;4:281–298. Reference Source

[ref-17] Guan D McCarthy SA Wood J : Identifying and removing haplotypic duplication in primary genome assemblies. *Bioinformatics.* 2020;36(9):2896–2898. 10.1093/bioinformatics/btaa025 31971576 PMC7203741

[ref-18] Gundappa MK Peñaloza C Regan T : Chromosome-level reference genome for European flat oyster ( *Ostrea edulis L.*). *Evol Appl.* 2022;15(11):1713–1729. 10.1111/eva.13460 36426132 PMC9679249

[ref-19] Gutiérrez-Zugasti I Andersen SH Araújo AC : Shell midden research in Atlantic Europe: State of the art, research problems and perspectives for the future. *Quat Int.* 2011;239(1–2):70–85. 10.1016/j.quaint.2011.02.031

[ref-20] Guy C Blight A Smyth D : The world is their oyster: Differences in epibiota on sympatric populations of native *Ostrea edulis* and non-native Crassostrea gigas ( *Magallana gigas*) oysters. *J Sea Res.* 2018;140:52–58. 10.1016/j.seares.2018.07.002

[ref-21] Haelters J Kerckhof F : Biodiversity Series: Background document for *Ostrea edulis* and *Ostrea edulis* beds. 2009.

[ref-22] Harry E : PretextView (Paired REad TEXTure Viewer): A desktop application for viewing pretext contact maps.2022; Accessed 19 October 2022. Reference Source

[ref-23] Helmer L Farrell P Hendy I : Active management is required to turn the tide for depleted *Ostrea edulis* stocks from the effects of overfishing, disease and invasive species. *Peer J.* 2019;7: e6431. 10.7717/peerj.6431 30842897 PMC6397756

[ref-24] Howe K Chow W Collins J : Significantly improving the quality of genome assemblies through curation. *GigaScience.* Oxford University Press.2021;10(1): giaa153. 10.1093/gigascience/giaa153 33420778 PMC7794651

[ref-25] Kerpedjiev P Abdennur N Lekschas F : HiGlass: web-based visual exploration and analysis of genome interaction maps. *Genome Biol.* 2018;19(1): 125. 10.1186/s13059-018-1486-1 30143029 PMC6109259

[ref-26] Lotze HK : Rise and fall of fishing and marine resource use in the Wadden Sea, southern North Sea. * Fish Res.* 2007;87(2–3):208–218. 10.1016/j.fishres.2006.12.009

[ref-27] Manni M Berkeley MR Seppey M : BUSCO update: Novel and streamlined workflows along with broader and deeper phylogenetic coverage for scoring of eukaryotic, prokaryotic, and viral genomes. *Mol Biol Evol.* 2021;38(10):4647–4654. 10.1093/molbev/msab199 34320186 PMC8476166

[ref-28] Perry F Jackson A : *Ostrea edulis* Native oysterIn: Tyler-Walters, H, Hiscock, K. (eds.) *Marine Life Information Network: Biology and Sensitivity Key Information Reviews*. Plymouth: Marine Biological Association of the United Kingdom,2017.

[ref-29] Philpots JR : Oysters, and all about them: being a complete history of the titular subject, exhaustive on all points of necessary and curious information from the earliest writers to those of the present time, with numerous additions, facts, and notes. 1891;2. Reference Source

[ref-30] Pogoda B : Current Status of European Oyster Decline and Restoration in Germany. *Humanities.* 2019;8(1):9. 10.3390/h8010009

[ref-31] Preston J Fabra M Helmer L : Interactions of larval dynamics and substrate preference have ecological significance for benthic biodiversity and *Ostrea edulis* Linnaeus, 1758 in the presence of *Crepidula fornicata.* * Aquat Conserv: Mar Freshw Ecosyst.* 2020;30(11):2133–2149. 10.1002/aqc.3446

[ref-32] Rao SSP Huntley MH Durand NC : A 3D map of the human genome at kilobase resolution reveals principles of chromatin looping. *Cell.* 2014;159(7):1665–1680. 10.1016/j.cell.2014.11.021 25497547 PMC5635824

[ref-33] Rhie A McCarthy SA Fedrigo O : Towards complete and error-free genome assemblies of all vertebrate species. *Nature.* 2021;592(7856):737–746. 10.1038/s41586-021-03451-0 33911273 PMC8081667

[ref-34] Rhie A Walenz BP Koren S : Merqury: Reference-free quality, completeness, and phasing assessment for genome assemblies. *Genome Biol.* 2020;21(1): 245. 10.1186/s13059-020-02134-9 32928274 PMC7488777

[ref-35] Rodriguez-Perez A James M Donnan DW : Conservation and restoration of a keystone species: Understanding the settlement preferences of the European oyster ( *Ostrea edulis* ). *Mar Pollut Bull.* 2019;138:312–321. 10.1016/j.marpolbul.2018.11.032 30660279

[ref-36] Simão FA Waterhouse RM Ioannidis P : BUSCO: assessing genome assembly and annotation completeness with single-copy orthologs. *Bioinformatics.* 2015;31(19):3210–3212. 10.1093/bioinformatics/btv351 26059717

[ref-37] South of England Oyster Company: Oysters and their cultivation.J. Wertheimer and co,1865. Reference Source

[ref-38] Surana P Muffato M Qi G : sanger-tol/readmapping: sanger-tol/readmapping v1.1.0 - Hebridean Black (1.1.0). *Zenodo.* 2023a; Accessed 21 July 2023. 10.5281/zenodo.7755665

[ref-39] Surana P Muffato M Sadasivan Baby C : sanger-tol/genomenote (v1.0.dev). *Zenodo.* 2023b; Accessed 21 July 2023. Reference Source

[ref-40] Thouzeau G Chauvaud L Durand G : Impact des polluants d’origine anthropique sur les organismes benthiques marins: notions d’indicateurs biologiques de perturbation et de réseaux de surveillance. In: *Académie des Sciences, actes du colloque RST Ingéniérie des Territoires, atelier “Science et aménagement des zones côtières, réflexions méthodologiques”*, Institut de FranceParis, Océanis 2003;27(2):177–214.

[ref-41] Uliano-Silva M Ferreira JGRN Krasheninnikova K : MitoHiFi: a python pipeline for mitochondrial genome assembly from PacBio high fidelity reads. *BMC Bioinformatics.* 2023;24(1): 288. 10.1186/s12859-023-05385-y 37464285 PMC10354987

[ref-42] Vasimuddin Md Misra S Li H : Efficient Architecture-Aware Acceleration of BWA-MEM for Multicore Systems.In: *2019 IEEE International Parallel and Distributed Processing Symposium (IPDPS).*IEEE,2019;314–324. 10.1109/IPDPS.2019.00041

[ref-43] Yonge CM : Oysters. Collins,1960.

[ref-44] Zhou C McCarthy SA Durbin R : YaHS: yet another Hi-C scaffolding tool. *Bioinformatics.* 2023;39(1): btac808. 10.1093/bioinformatics/btac808 36525368 PMC9848053

[ref-46] Wellcome Sanger Institute: The genome sequence of the European flat oyster, *Ostrea edulis* (Linnaeus, 1758). European Nucleotide Archive[dataset], accession number PRJEB57260.2022.

[ref-45] Zu Ermgassen PSE Spalding MD Blake B : Historical ecology with real numbers: past and present extent and biomass of an imperilled estuarine habitat. *Proc Biol Sci.* 2012;279(1742):3393–3400. 10.1098/rspb.2012.0313 22696522 PMC3396889

