# Supplementary material for: The genome sequence of the European flat oyster, Ostrea edulis (Linnaeus, 1758)
Source: Wellcome Open Res. 2023 Dec 5;8:556. [Version 1] doi: 10.12688/wellcomeopenres.19916.1 (PMC10979132; doi:10.12688/wellcomeopenres.19916.1)
Supplement: Peer review history

# Reviewer report: Reviewer response for version 1

**DOI:** 10.21956/wellcomeopenres.22053.r76947

**Published:** 2024-03-28

**Authors:** Miguel Naranjo-Ortiz

**Affiliations:** 1 University of Oslo, Oslo, Norway

The methodology is solid and the results are well presented and accessible.

 There are two aspects I believe would enhance the paper. These comments are just a suggestion and not a requirement:

 The first is providing a more comprehensive description of the mitochondrial genome. The mitochondrial genome has been widely studied in mollusks, so it is a point of interest in this group in particular.

 The second is a comparison with the previous reference genome for the species. While I understand that this assembly is of a much higher quality, having two independent assemblies can provide a good view into the intraspecific genomic diversity of the species.

Are sufficient details of methods and materials provided to allow replication by others?

Yes

Is the rationale for creating the dataset(s) clearly described?

Yes

Are the datasets clearly presented in a useable and accessible format?

Yes

Are the protocols appropriate and is the work technically sound?

Yes

Reviewer Expertise:

Comparative genomics, evolutionary genomics, mycology

I confirm that I have read this submission and believe that I have an appropriate level of expertise to confirm that it is of an acceptable scientific standard.

# Reviewer report: Reviewer response for version 1

**DOI:** 10.21956/wellcomeopenres.22053.r75505

**Published:** 2024-03-27

**Authors:** Tim P. Bean

**Affiliations:** 1 The Roslin Institute and Royal (Dick) School of Veterinary Studies, University of Edinburgh, Midlothian, UK

This paper represents a new high-quality genomic resource for the European flat oyster *Ostrea edulis*. All the data required is provided and the methods used to generate it are clearly described. This paper and associated genome data will provide an excellent resource for the research and conservation communities. I have just a few minor comments below...

- Native oysters have highly variable shell colour. Maybe remove reference to colouration.
- “700 million oysters caught in London”. Should this say *eaten* in London?
- I can’t find evidence to support the statement that *M. gigas* has replaced *O. edulis* (within the cited Haelters paper). Maybe remove this statement.

Are sufficient details of methods and materials provided to allow replication by others?

Yes

Is the rationale for creating the dataset(s) clearly described?

Yes

Are the datasets clearly presented in a useable and accessible format?

Yes

Are the protocols appropriate and is the work technically sound?

Yes

Reviewer Expertise:

Oyster genomics, genetics and disease.

I confirm that I have read this submission and believe that I have an appropriate level of expertise to confirm that it is of an acceptable scientific standard.
